# Making Sense of Developmental Kinetics Under High-Sugar Stress: Mathematical Modeling of Phenotypic Plasticity in *Drosophila melanogaster*

**DOI:** 10.3390/nu18081255

**Published:** 2026-04-16

**Authors:** Bence Pecsenye, Maha Rockaya, Tünde Pacza, Zibuyile Mposula, Endre Máthé

**Affiliations:** 1 Institute of Nutrition Science, Faculty of Agricultural and Food Sciences and Environmental Management, University of Debrecen, Böszörményi Str. 128, H-4032 Debrecen, Hungary; maha.rockaya@agr.unideb.hu (M.R.); pacza.tunde@unideb.hu (T.P.); zibuyile.angel@agr.unideb.hu (Z.M.); 2 Doctoral School of Food Science, Faculty of Agricultural and Food Sciences and Environmental Management, University of Debrecen, Böszörményi Str. 128, H-4032 Debrecen, Hungary; 3 Department of Life Sciences, Faculty of Medicine, Vasile Goldis Western University of Arad, L. Rebreanu Str. 86, RO-310414 Arad, Romania

**Keywords:** *Drosophila melanogaster*, nutritional modeling, sugar stress, sucrose, life cycle, developmental kinetics

## Abstract

**Background/Objectives:** Although *Drosophila melanogaster* is widely used in genetics and nutrition research, developmental kinetics are rarely analyzed using formal mathematical modeling. Most dietary studies present developmental curves without rigorous fitting, limiting quantitative interpretation. This study applies and compares three primary models, as well as develops secondary models, to characterize the effects of high-sugar diets on egg-to-adult (life cycle) development. **Methods:** Standardized husbandry and an embryo-to-pupa feeding assay were performed across 11 sucrose concentrations. Synchronized embryo collection and high-resolution monitoring were used for this assay. Three primary models—dose–response, Gompertz, and logit-based linearization—were fitted to developmental curves to extract timing (***t_mid_***) and synchrony (***s_dvp_***) parameters. Secondary modeling was used to evaluate how these parameters change with respect to sucrose concentration. **Results:** Increasing sucrose concentration markedly delayed pupariation and reduced viability at the highest levels. All models showed increasing ***t_mid_*** and decreasing ***s_dvp_*** with rising sugar concentration, with the Gompertz model providing the best overall performance. Secondary modeling revealed a consistent bilinear response with a breakpoint at 0.52–0.62 M, separating low-, medium-, and high-sucrose conditions. Reduced sampling frequency decreased model robustness, while twice-daily observations remained sufficient. **Conclusions:** Mathematical modeling provides a robust, practical framework for quantifying the effects of diet on *D. melanogaster* development. The Gompertz model provided the best fit and yielded biologically interpretable parameters. The bilinear secondary model effectively captured sucrose-dependent stress responses and quantified plasticity through environment-dependent changes in developmental timing and synchrony. Overall, this work establishes a quantitative practical framework for modeling developmental kinetics under nutritional perturbations, and the approach can be extended with additional secondary environmental factors to improve predictive analyses of nutritional effects.

## 1. Introduction

*Drosophila melanogaster* as a model animal goes back over a century, the first scientific paper of what was at the time called *Drosophila ampelophila* as an experimental organism was published in 1905 [1]. Since then, it has experienced two golden age periods of research [2]. The first was as a tool to introduce the field of transmission genetics in the 1910s, carried out by Thomas Hunt Morgan and his colleagues, with the first convincing evidence of sex-linked inheritance in animals through the discovery of the white-eye mutation. *D. melanogaster* proved to be an excellent animal to study mutations, in no small part because of its short generation time as well as the large number of offspring, producing many spontaneous mutants. The second golden age period was in the 1970s, when the fruit fly became a tool to study the genetic control of otherwise complex and hard-to-study biological processes [2]. Nowadays, the fruit fly is the central model organism in many fields, including genetics, metabolism, developmental biology, and aging, due to it being the first complex multicellular organism to have its full genome sequenced, and the existence of large “fly banks” (BDSC, KDC, etc.) that maintain a large number of mutant strains available for researchers to study. It also has a big role in the study of human diseases and nutrition, since 60–75% of genes associated with those diseases have fly orthologs [3,4].

As far as nutrition is concerned, our main area of interest is feeding experiments on larvae and adult flies. There is a plethora of recent research papers conducting different feeding experiments on larval stages and, in general, the embryo-to-adult life stages [5,6,7,8,9,10,11], but to our knowledge there is no unified and established modeling done on the kinetics of development. Most studies that conduct these experiments show developmental curves with no- or undisclosed fitting, presenting these curves accompanied by EAV (embryo-to-adult viability), mean pupation/eclosion time—all of these could be strongly improved by mathematical modeling to grant a more accurate result and characterize the kinetics of development with a higher number of parameters. One exception we found is Havula et al. [6], where they used a sigmoid fit function in the sicegar package in R, which uses the three-parameter logistic function [12], but only to extract the mean pupation time parameter. There is also a need to improve the accuracy of these experimental results by increasing the resolution of the raw data, but to our knowledge, no studies have tried to optimize experimental design to achieve this. To be able to compare results from an ever-growing number of different studies, there is a need to provide accurate quantitative data, with an inevitable future of using databases to handle the ever-increasing amount of laboratory data.

Although when it comes to *D. melanogaster* developmental kinetics, mathematical modeling is not yet a requirement, in many other fields it is the standard. Such attempts have been made, for example, when it comes to the longevity and aging of *D. melanogaster* [13,14], and to growth and maturation when it comes to larval weight and pupation timing [15]. Outside of experiments on fruit flies, in the fields of microbiology, pharmacokinetics and many other fields of the life sciences, kinetics modeling is at a much more advanced stage [16,17].

We also noticed in the literature that flies reared on some high-sugar diets (HSDs) not only caused a delay (lengthening of larval stages) but also reduced uniformity of the development of such individuals. As an example, one paper by Musselman et al. (2011) studying obesity and insulin resistance on HSDs, included some form of curve fitting; however, the undisclosed model they used completely ignored this effect—most visible in their high-sucrose diet—although developmental delay was mentioned, it was not the focus of their research [11].

In this study, we focused on the effects of a high-sugar diet (HSD) on the developmental kinetics of *Drosophila melanogaster*, since it has been a subject of much research for its ability to induce obesity, insulin resistance and type 2 diabetes in fruit flies [6,11,18,19]. On top of that, it has been well established that such diet acts as a stressor for developing larvae as well as adult flies [18,20], causing drastic delays and lengthening the larval stages by up to several days [6,10,11]. To our knowledge, this is a pilot study implementing primary mathematical models to describe the egg-to-adult development of *D. melanogaster* and secondary models to describe the effects of HSDs.

## 2. Materials and Methods

Following the terminology of Rockaya et al. [21], the methods are divided into “wet” and “dry.” The “wet” refers to experimental procedures performed at the laboratory bench, including all associated materials and equipment. In contrast, “dry” refers to computer-based mathematical or statistical analyses performed at an office bench.

### 2.1. “Wet” Methods

#### 2.1.1. Fruit Fly Husbandry

The experiment was carried out on the *w^m4h^* mutant strain (Bloomington Drosophila Stock Center, Bloomington, IN, USA). *Drosophila melanogaster* stock was maintained on culture media containing 70 g/L yeast paste (Lesaffre Magyarország Kft., Budapest, Hungary), 0.15 M sucrose (51.3 g/L), (VWR International, Debrecen, Hungary), 30 g/L semolina (Sam Mills International, Botiz, Romania), 10 g/L agar (VWR International, Debrecen, Hungary) and 1 g/L Nipagin (Thermo Fisher Scientific, Waltham, MA, USA). The stock was kept at 25 °C and transferred to new bottles with fresh culture media every two weeks.

#### 2.1.2. Embryo Harvesting and Feeding Experiment

To acquire fertile young adult flies for embryo collection, flies were transferred to fresh bottles and left to oviposit for five days, then removed from the bottles. The first few days of freshly emerged flies were removed. Then, two days later, the young 0–2 days old flies were moved to fresh media yet again and left to mature for three days.

**Figure 1:**
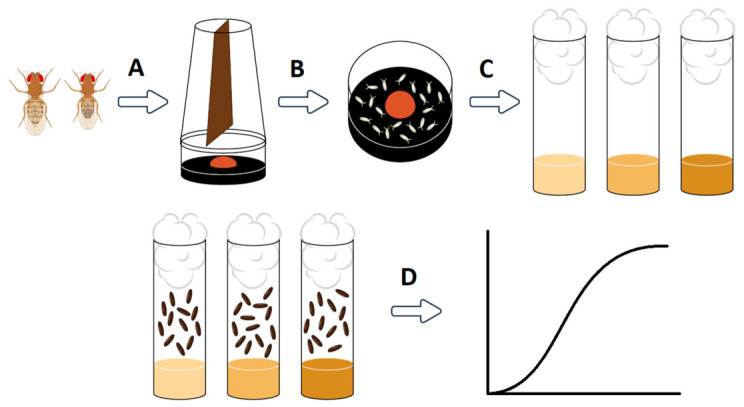
A schematic flowchart of the experiment: (**A**) Placing pairs of 3–5-day old adults into an embryo collection cage; (**B**) Acquiring Petri dish full of fresh synchronized embryos; (**C**) Collection of embryos into vials containing sample media; (**D**) Marking new pupae every eight hours to gather data for analysis of kinetics curves.

Around 400 of these 3–5 days old individuals were placed into an egg collection cage—a plastic cylinder with a rough mesh breathable top, and a plate across the middle to provide sufficient surface area. Under the cage, a Petri dish with 1% agar, darkened with 1% activated charcoal [22], was placed to serve as the oviposition surface. In the middle of the plate, a thick 2 cm radius circle of yeast–water mixture was spread, with a consistency of peanut butter, as a food source for the parent flies and left overnight (Figure 1A). The next morning, the Petri dish was replaced with a fresh one, but with a much smaller circle of yeast–water mixture to encourage oviposition [23]. This was repeated two more times every 2 h, each time discarding the Petri dish of embryos, and only the third Petri dish was kept and used for embryo collection (Figure 1B). This was done to ensure that the embryos are sufficiently age-synchronized to a 2 h period by getting rid of retained embryos [24]. This type of synchronization is expected to produce syncytial blastoderm-type cleavage-stage embryos without cellular blastoderm, which is specific to *Drosophila* early embryogenesis. The embryos were collected under stereomicroscope by hand using blunted forceps to avoid damaging them (Figure 1C). To obtain high-resolution data while avoiding the larval crowding effect, 50 embryos were counted into each test tube containing 7 mL of sample media [25]. Sample media were prepared as described above, but with 11 different sucrose concentrations: 0 M–1.5 M in 0.15 M increments. Sucrose was selected for its ability to elicit a potent stress response, as well as its proven effectiveness as a powerful attractant for both larvae and adults [11,26,27]. Five replicates were prepared for each concentration (*n* = 250 embryos) and kept at 25 °C in an incubator. Throughout the entire experiment, the test tubes were monitored daily. Data collection began when the first pupae hatched and continued every eight hours to increase data resolution and investigate the effect of sampling frequency on accuracy. A marker pen was used to mark each newly formed pupae on the vials, and then the number of pupae was counted. The newly eclosed adult flies were then removed and counted (Figure 1D).

### 2.2. “Dry” Methods

This section introduces the primary and secondary models adapted from predictive microbiology and applied to fit the developmental data of *Drosophila melanogaster* [28].

#### 2.2.1. Primary Modeling

Primary growth models describe the change in *Drosophila melanogaster* population over time under constant environmental conditions, i.e., the number of hatched flies as a function of time. In our case, for each sugar concentration, a single growth curve was obtained based on five replicates, which was then fitted with a primary model to extract a set of parameters—commonly referred to as primary parameters.

In what follows, we present the primary models used in this study:

##### Dose–Response Function

The dose–response (DR) function is commonly used in pharmacology and toxicology to relate a quantitative response to an increasing dose of a treatment. In developmental studies, it provides a flexible sigmoidal curve capable of describing monotonic transitions, such as the progression from low to high numbers of hatched flies over time. However, our data are always normalized; thus, the progression will be described by the ratio ***y_obs_***/***y_max_*** (which takes values from 0 to 1) as a function of time. The steepness of the transition and the time required to reach 50% of the maximum response are explicitly parameterized, making this function convenient for comparing developmental synchrony across treatments.

The specific functional form applied here is presented in Equation (1):

(1) $$y\left(t\right)=\frac{y_{max}}{1+10^{-s_{dvp}\left(log(t\right)\;-\;log(t_{mid}))}}$$

where

***s_dvp_*** is the synchrony parameter.

***t_mid_*** is the mean pupariation or eclosion time, which is the average time required to reach 50% of the maximum response. Since our data are normalized, it is the time needed to reach ***y*** = 0.5.

***y_max_*** is the maximum viability % of the individuals under each constant condition. When the curve is normalized by the final number of pupae/hatched flies at the end of the experiment, this value is fixed to 1.

##### Gompertz Function

The original Gompertz model was first introduced to describe the time variation in population size. Since then, it has been widely applied in diverse biological contexts, including tumor growth and the natural logarithm of bacterial concentration [29]. In predictive microbiology and developmental studies [17], its asymmetric sigmoid shape makes it suitable for modeling developmental processes with a clear lag, growth, and plateau phase.

The functional form used in this study is shown in Equation (2):

(2) $$y\left(t\right)=y_{0}+\left(y_{max}-y_{0}\right)\cdot exp\left(-exp\left(\frac{s_{dvp}}{y_{max}-y_{0}}\cdot e\cdot (\lambda -t)+1\right)\right)$$

where

***y*_0_** is the initial number of pupae or hatched flies at time ***t*_0_**. In our experiment, this value was logically set to zero because observations began at embryo collection, and pupariation did not occur for several days.

***λ*** is the lag parameter defined as the time (in hours or days) required before the first pupariation or eclosion. In other words, ***λ*** represents the shift in the sigmoid curve on the *x*-axis.

***s_dvp_*** is the synchrony parameter, in this model referring to the maximum developmental rate of the fly/larva population (1/time). It represents the steepest slope of the fitted growth curve, i.e., the maximum number of pupae or emerged flies produced per unit time at the curve’s inflection point.

***y_max_*** is the maximum viability, it was explained before in the Dose–Response function section.

As mentioned before, ***y*_0_** = 0 (in all cases) and ***y_max_*** = 1 (for normalized data). After applying these, the equation becomes Equation (3):

(3) $$y\left(t\right)=exp(-exp\left(s_{dvp}\cdot e\cdot (\lambda -t)+1\right))$$

Although this function does not have ***t_mid_*** parameter, this value can be obtained by making ***y*(*t*) = *y_max_*/2** (***y*(*t*) = 0.5** for normalized data), rearranging it and solving it for the time variable ***t*** to get ***t_mid_***.

##### Logit Function

The logit function is commonly used to linearize sigmoidal growth curves and stabilize variance prior to statistical modeling. It normalizes the data by using the ratio of the observed response to its maximum possible value ***y_obs_***/***y_max_***. The logit function then converts bounded proportional data (i.e., a variable with a value only between 0 and 1) into an unbounded scale (–∞, +∞), it also makes the data more symmetric around the mean and stabilizes the variance. All of these make it suitable for linear regression, which is the holy grail of modeling. From the linear function, we can extract ***t_mid_*** (*x*-intercept) ***s_dvp_*** (slope).

The logit transformation (rescaling) used in this study is given in Equation (4):

(4) $$y_{logit}=Ln\left[\frac{\frac{y_{obs}}{y_{max}}}{1-\frac{y_{obs}}{y_{max}}}\right]$$

Then, we fit the new variable against time using a simple linear model.

#### 2.2.2. Secondary Modeling

Secondary models describe how the parameters of a primary model change as a function of environmental conditions (sugar concentration, in our case). The ***s_dvp_*** and ***t_mid_*** estimates obtained from the primary fitting (see Section 2.2.1) were regressed against 11 different sugar concentrations. Treating these parameters as derived phenotypic traits, their environmental dependence was considered. These were interpreted as reaction norms, with ***t_mid_*** (E) representing the reaction norm of mean developmental timing and ***s_dvp_*** (E) representing the reaction norm of developmental synchrony. Phenotypic plasticity was thus quantified as the change in these parameters across environments (E)—which, in our case, was sucrose concentration [30].

Two different models were used based on the trend of the relationship that emerges from plotting the estimates against sugar concentrations:

##### Linear Model

A linear model is selected when the parameter displays a consistent, monotonic change with sugar concentration, indicating that a single linear relationship adequately captures the trend.

(5) y=a·x+b

where *x* is the *x*-axis value, ***a*** is the slope of the fitted line and, ***b*** is its intercept with the *y*-axis.

##### Bilinear Model

A bilinear model is used when the relationship between the parameter and sugar concentration shows a distinct change in slope, suggesting two linear phases separated by a critical transition point.

(6) $$y_{1}=a_{1}\cdot x+b_{1}\;\mathrm{if}\;x\leq x_{c}$$

(7) $$y_{2}=a_{2}\cdot x+b_{2}\;\mathrm{if}\;x>x_{c}$$

***x_c_*** is the break/critical point between the two linear segments of the model, which can be calculated by:

(8) $$y_{1\left(c\right)}=y_{2(c)}\to \;a_{1}\cdot x_{c}+b_{1}=a_{2}\cdot x_{c}+b_{2}\to \;x_{c}=\frac{a_{1}-a_{2}}{b_{2}-b_{1}}$$

where ***a*_1_** and ***a*_2_** are the slopes of the two linear segments of the model, and ***b*_1_** and ***b*_2_** are their intercepts with the *y*-axis.

#### 2.2.3. Model Fitting and Statistical Analysis

The fitting of primary and secondary models was done using the Frontline Systems Inc. (Incline Village, NV, USA) Solver Add-in for Microsoft Excel Version 16.0 (Microsoft 365) (Redmond, WA, USA) with the GRG Nonlinear to perform nonlinear least squares fitting.

ANOVA and Tukey’s test are used to check the significance of the difference between the viabilities (***y_max_***) across the different sugar concentrations [31,32].

The Akaike Information Criterion based on SSE was applied when comparing secondary models [33].

Levene’s approach was used for testing homoscedasticity [34].

## 3. Results

**Figure 2:**
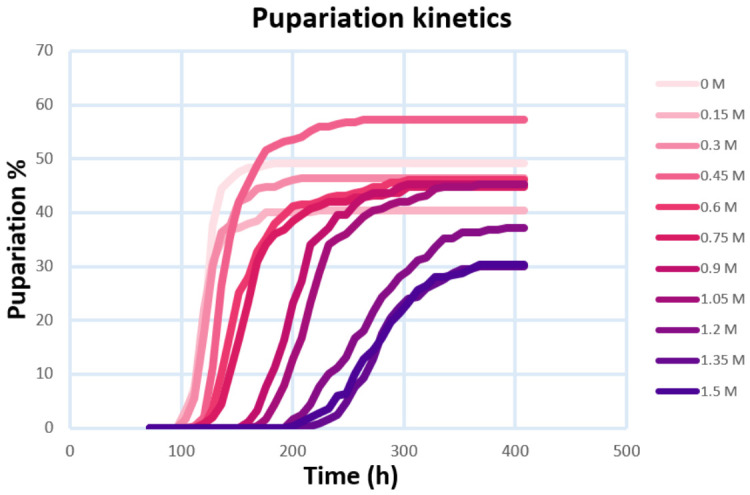
Pupariation kinetics curves without fitting, using an average of five replicate vials (*n* = 250 individuals). Pupariation % refers to the proportion of initial embryos reaching pupation.

The following results are demonstrated on the pupariation kinetics of our experiments, but in a similar fashion, they can be applied to EAV (embryo-to-adult viability) kinetics as well. After data acquisition, we can determine a few qualitative things, including the effect of the studied diets or additives on the developmental curves, which could include a notable difference in the length of larval stages when compared to a control experiment or between different diets. In our case, this comparison we made between different sucrose content in the fly media (low- to high-sugar diets). On lower-sucrose content diets, larval stages were shorter—first pupae were noted as early as 96 h from oviposition and the last at 184 h—while as sugar concentration increased, this was delayed by over 100 h (Figure 2).

**Figure 3:**
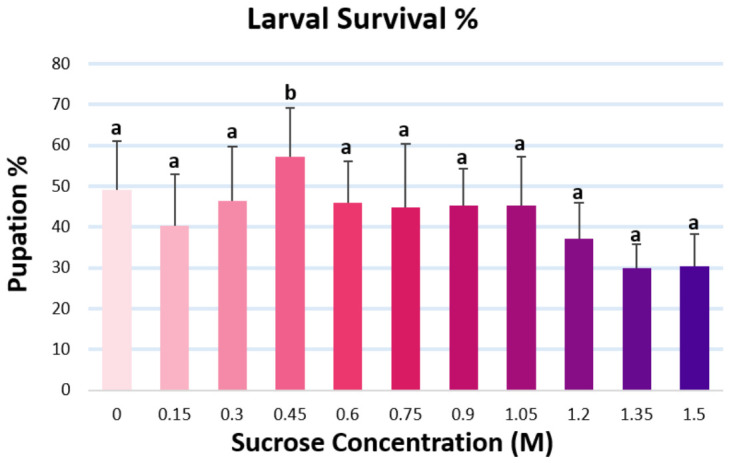
Larval survival represented by % of individuals reaching pupa stage from *n* = 250 individuals per sucrose concentration. Error bars represent standard deviation values, and letters correspond to groups assigned by Tukey’s test following one-way ANOVA, indicating that bars with the same letter are not statistically significantly different, while bars not sharing the same letter are statistically significantly different at *α* = 0.05.

In addition to these observations, we can also make quantitative determinations when it comes to viability (survival %) on different diets. Our experiments did show a decrease in larval survival as the sugar concentration increased. ANOVA showed a statistical difference (*p* = 0.0153) between the sugar concentrations with a large effect size (*η^2^* = 0.37). To find the exact source of the difference, we ran Tukey’s test to do pair-wise significance checks, and it only showed statistically significant differences between 0.45 M to 1.35 M (*p* = 0.0149) and 0.45 M to 1.5 M (*p* = 0.0174) (Figure 3). To test our assumptions, normality of residuals was evaluated graphically. The residuals showed no substantial deviation from normality, with a skewness of −0.03 and kurtosis of −0.51. For testing homoscedasticity, Levene’s approach was used, showing no significant heterogeneity (*p* = 0.7091) based on absolute deviations from group means.

While these results are in and of themselves valuable, mathematical modeling can turn these qualitative observations into reliable quantitative data.

### 3.1. Primary Fitting and Model Performance

**Figure 4:**
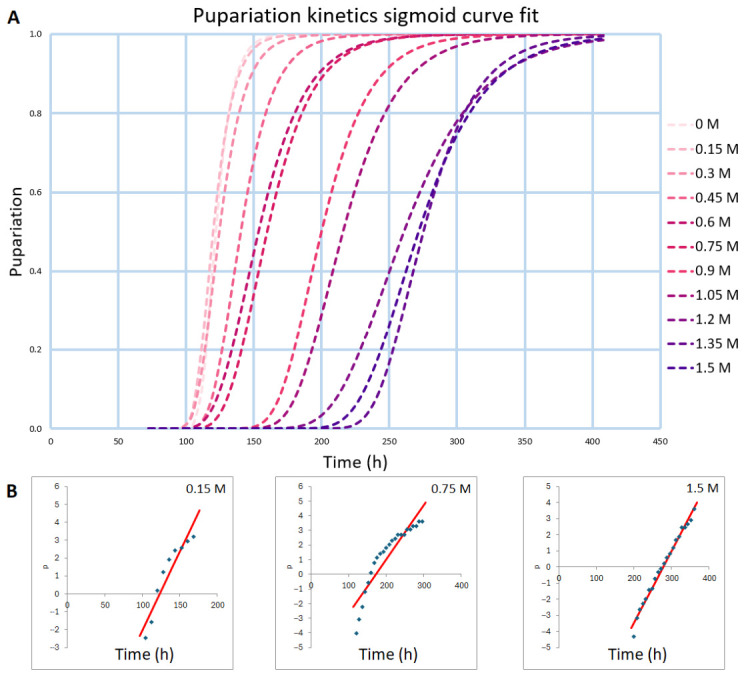
Example primary fits: (**A**) Sigmoidal functions (Gompertz, Dose–Response); (**B**) Logit function transformation with linear regression.

For primary fitting, all developmental curves were reparametrized from ***y_obs_*** to ***y_obs_***/***y_max_***, yielding normalized curves with ***y_obs_***/***y_max_*** values between 0 and 1. After normalizing our data and fitting using the functions (Figure 4 and Figure S1), as described in Section 2.2, we can extract the ***t_mid_*** and ***s_dvp_*** parameters, by which we can characterize our experiments’ developmental curves (Table 1 and Table 2). All three functions used can describe our dataset satisfactorily, but each of them has some advantages and disadvantages.

The dose–response function’s synchrony (***s_dvp_***) parameter—although it correlates with the slope—does not describe the maximum developmental rate of the fly/larva population over time, therefore, in our opinion, it is a less useful parameter when compared to the other two functions’ respective synchrony parameters.

The logit function “stretches” the sigmoid-shaped curve, allowing linear regression to be used, which is very desirable when it comes to mathematical modeling—generally, the simplest function is the most desired—however, extracting the slope and the *x*-intercept of the linear fit requires an additional step.

In the case of the Gompertz function, as described above in the Gompertz Function section, getting the ***t_mid_*** parameter is a little more time-consuming.

Using all three functions, the ***t_mid_*** parameter shows a positive correlation—meaning increased delay—with sugar concentration, while the ***s_dvp_*** parameter shows negative correlation—meaning less uniformity between individuals—with sugar concentration (Table 1 and Table 2). The investigation of these relations is a matter of secondary modeling.

### 3.2. Secondary Fitting and Model Performance

**Figure 5:**
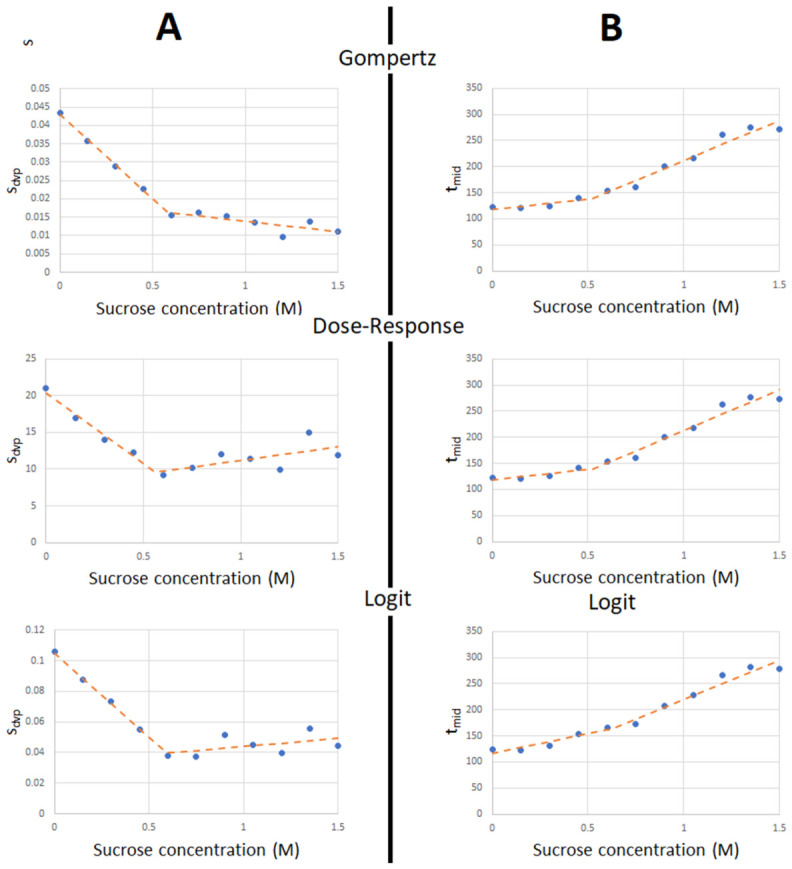
Secondary fits (dotted line) using bilinear function on primary parameter values across sucrose concentrations (dots): (**A**) Comparison of sucrose concentration—***s_dvp_*** graphs using all three primary functions; (**B**) Comparison of sucrose concentration—***t_mid_*** graphs using all three primary functions.

As shown in Figure 5A, when we graph synchrony as a function of sucrose concentration, we can, in all three cases, notice a pattern that emerges. This pattern seems to be a strong negative linear correlation at lower sugar concentrations and a weak linear correlation that is negative in the case of the Gompertz function and positive in the other two cases. Fitting a bilinear function describes this relation quite well, with a breakpoint (***x*_c_**) concentration that is shown in Table 3.

When it comes to the timing parameter, we still see a bilinear pattern—though a much weaker one—that is very similar across all three primary functions. The breakpoint in this case, as in the previous one, is at around the same concentrations, suggesting that these stress effects, which manifest in a delay and lowered synchrony in development, may be caused by the same source.

R^2^ values of the bilinear fit for ***s_dvp_*** are highest when using the Gompertz primary function, but for the ***t_mid_*** parameter, the R^2^ values are similar in all cases. Overall, the best fit is gained by using the Gompertz function, as shown by the Normalized Root Mean Square Error (nRMSE) (Table 3).

### 3.3. Sampling Frequency Effect on Parameters and Residuals

To avoid overfitting, we compared the Akaike Information Criterion (*AIC*) values for linear and bilinear fits. *AIC* values were consistently lower for the bilinear fits and Δ*AIC* > 2 was true for all models and parameters, supporting the use of bilinear fitting even after accounting for a higher number of parameters (4 for bilinear—2 for linear) (Table 4).

Confidence intervals for the bilinear model were estimated using residual bootstrap resampling (*n* = 1000) on the Gompertz primary model parameter-based secondary models. For the sucrose concentration—***s_dvp_***, the estimated breakpoint was ***x_c_*** = 0.581 M, with a 95% confidence interval of 0.505 to 0.665 M. The intercept was estimated at 0.0427 (95% CI: 0.0408 to 0.0447), the slope before the ***x_c_*** at −0.0466 (95% CI: −0.0536 to −0.0396), and after ***x_c_*** at −0.0053 (95% CI: −0.0089 to −0.0016). For the sucrose concentration—***t_mid_*** the estimated breakpoint was ***x_c_*** = 0.524 M, with a 95% confidence interval of 0.210–0.970 M. These results show a well-defined breakpoint for the sucrose concentration—***s_dvp_*** bilinear model, but a very wide range for the sucrose concentration—***t_mid_***. Although both parameters suggest a transition occurring in a similar concentration range and model selection (AIC) favors the bilinear formulation, the wide confidence interval for ***t_mid_*** indicates that its breakpoint estimate is less robust and more sensitive to data perturbations.

To demonstrate the effects of sampling frequency, based on our results, we used the Gompertz primary function with a bilinear secondary function. Since most publications used a sampling frequency of 24 h, and data acquisition outside of work hours might be a problem for some laboratories, we used our dataset to investigate how reducing the number of observations affects parameters, residuals and R^2^ of fit. We compared the previously used 8 h interval to twice a day (9 a.m., 5 p.m.) and once a day (9 a.m.). Our findings are shown below (Table 5).

We can see that the breakpoint does not change significantly when going from three to two observations per day but does change significantly when going from two to one observation per day in the case of the sucrose concentration—***s_dvp_*** bilinear fit, but does not change much in the case of the timing parameter. For residuals, decreasing sampling frequency deteriorates the fit, increasing nRMSE. Generally, the steeper the curve, the more observations are recommended, since in stressed environments it could take several days to reach the endpoint (like in our case at the higher sucrose concentrations), which ensures that even with once-a-day observation, there will be plenty of data points for a good fit.

## 4. Discussion

In this study, we examined the effects that diet has on *Drosophila melanogaster* larval development. Since previous studies have shown significant variation in developmental kinetics depending on sugar content (most significantly sucrose) [5,26], with HSD causing delays in larval development, potentially linked to reduced intestinal digestion and nutrient absorption [35,36]. In addition, experiments on high-sugar diet play an important role in medical and nutritional research, not only for their effectiveness in inducing obesity, insulin resistance and type 2 diabetes, but also for modeling the high-carbohydrate diet commonly consumed by large human populations in the 21st century [6,11,18,19,37,38,39].

We identified a methodological gap in current research, as most *Drosophila melanogaster* developmental studies report developmental outcomes descriptively, as opposed to by clear quantitative data that could be derived by mathematical modeling. Modeling developmental kinetics can help produce reliable quantitative data, making progress in predictive nutrition, as well as assisting in addressing the yet-unsolved problem of holidic diets (what combination of macro- and micronutrients would present similar developmental kinetics to yeast-based media) for *D. melanogaster* [40]. We outlined a simple experimental protocol that is optimized for studying and quantifying the effects of nutrition on larval stages for the purposes of simplifying the process of modeling, but can be extended, as described above, up to eclosion.

We compared three commonly used sigmoidal functions to describe the kinetics curves of *D. melanogaster* development, namely Gompertz, Dose–response, and Logit. We outlined in Section 3 the advantages and disadvantages of each method for extracting biologically meaningful parameters of viability (***y_max_***), developmental synchrony (***s_dvp_***) and mean developmental timing (***t_mid_***). These parameters are useful tools for a couple of reasons: 1—providing quantitative descriptors of dietary stress on development caused by diet; 2—offering easy-to-understand quantitative data for use in databases or for comparison with results in the literature; 3—providing a fast, cheap, easy and effective means to test if a studied drug, food additive, or type of diet can normalize these parameters to their optimal (control) values, indicating a potential rescue effect from stress caused by an environmental factor (HSD in our case).

Secondary modeling of these parameters for the environmental factor of sucrose content presented a clear bilinear response pattern in both ***t_mid_*** and ***s_dvp_*** parameters. Interpreting these secondary functions as reaction norms clarifies the biological meaning of this pattern: ***t_mid_*** (*E*) represents the reaction norm of mean developmental timing, describing phenotypic plasticity in developmental timing, whereas ***s_dvp_*** (*E*) represents the reaction norm of developmental synchrony, reflecting changes in the spread of individual developmental times. Below the breakpoint, both parameters changed steeply with increasing sucrose, indicating strong plasticity in both timing and synchrony; above this threshold, parameter values plateaued, suggesting that additional dietary sugar produced little further change in developmental kinetics and that stress effects were near maximal for these traits, while viability continued to decrease. The results can be interpreted using the concept of canalization, defined as the tendency of developmental systems to produce consistent phenotypes, resisting the effects of genetic and environmental perturbations, as well as the concept of phenotypic plasticity, which describes systematic shifts in trait values across environments that are represented by reaction norms. Therefore, increased temporal dispersion quantified by reduced ***s_dvp_*** values with increasing sucrose concentration, is a classic example of stress-induced decanalization, where weakened developmental buffering exposes latent variability between individual flies [40]. Bilinear fitting identified a critical point (breakpoint) where this pattern switches from one linear correlation to another. We found this critical sucrose concentration to be around 0.52 M to 0.62 M. Above this threshold, we would consider the diet to be HSD, since the timing and synchrony parameters change very little above this breakpoint, indicating that sugar stress is close to maximal when it comes to affecting developmental kinetics. This value of around 20% sucrose coincides with previous literature, where they commonly use media containing sugar above this concentration when describing HSD [7,11,18,27,41,42]. At lower sucrose concentrations, the developmental kinetics parameters appear to respond strongly to increased dietary sugar availability. After the breakpoint, this response begins to plateau, which suggests saturation of physiological processes. This behavior is consistent with threshold-like responses in nutrient-sensing systems, where post-ingestive detection of nutrients trigger endocrine and metabolic adjustments. Recent work has emphasized that nutrient sensing is mediated by integrated organ system inputs, including gut-derived signaling, insulin-like pathways and central nervous system inputs, which regulate growth and metabolism in response to dietary conditions [43]. The emergence of a breakpoint in the above sucrose concentration range may reflect a transition region where these nutrient-sensing pathways approach maximal activation, leading to a diminishing response that manifests in changes in developmental timing and synchrony despite further increases in sucrose concentration.

Although we used a sampling frequency of 8 h, and for more accurate parameters we recommend this frequency or higher. There have been efforts to develop more accurate automated detection methods for sensing developmental stage changes [44], but these still have relatively small throughput. We examined the effect of decreasing frequency to twice a day or once a day. Our results show that for the timing parameter, even once a day could be sufficient; however, for the synchrony parameter, this is only enough under higher-stress diets. In culture conditions or low-stress environments, once-a-day sampling provides as few as two to three points of useful data, making fitting inaccurate. Some laboratories might have difficulty sampling outside of work hours, but even those labs can usually achieve morning and afternoon data collection. With these timeslots (9 a.m., 5 p.m.), our results were comparable to three-times-a-day data collection; therefore, we recommend sampling at this frequency if higher is not possible.

Lastly, this study could be considered as a first step in modeling developmental kinetics. Further research needs to be done to include more environmental factors in the secondary model. These should mainly be other macronutrients (especially protein), temperature (although single-factor temperature models have been attempted [45]), and water activity/water content of media. It is important to study multiple factors that could possibly have a combined effect on development, such as sugar content and water content [46], or sugar content and antioxidant activity.

## 5. Conclusions

*Drosophila melanogaster* remains an exceptionally powerful model organism for nutritional science, owing to its short generation time, well-annotated genome, extensive developmental and tissue-specific transcriptome and interactome sources, extensive genetic resources, and strong conservation of diet- and disease-related pathways with humans. The experimental design outlined in the current study provides a solid practical framework. It uses tightly synchronized, genetically and epigenetically identical fruit fly individuals that are exposed to defined nutritional interventions throughout their entire life cycle. These interventions start from the early embryonic stage and continue through the hatching of larvae and the eclosion of adults from the pupal case. In addition to nutrition and minimizing larval crowding, environmental parameters such as humidity, ambient temperature, and diurnal cycles can be controlled and monitored. The aforementioned features of the *Drosophila*-based model provide the most comprehensive nutritional assessment available and, as such, appear to be more advanced than human evaluations. Surprisingly, the *Drosophila* model deals with far fewer variables that would complicate the interpretation of experimental data.

This study demonstrates the versatility and reliability of the *Drosophila*-based nutritional assessment by introducing and comparing three primary sigmoidal models and developing secondary models that relate developmental parameters to sucrose concentration. Mathematical modeling substantially enhances the reliability and comparability of developmental data while providing a richer set of quantitative descriptors through primary parameters.

We demonstrate that high-sugar diets induce substantial developmental delay and reduced developmental synchrony, and that these effects follow a consistent bilinear pattern with a critical sucrose threshold concentration between 0.52 and 0.62 M, aligned with concentrations widely used to define high-sugar diets in the literature. The convergence of breakpoints across all three primary models suggests that these values are unlikely to be mathematical artifacts but instead reflect an underlying biological transition. Among the tested models, the Gompertz function yielded the best overall fit and enabled robust quantitative characterization of both developmental delay and developmental synchrony (dispersion). Furthermore, our analysis highlights the importance of high sampling frequency, with twice-daily measurements offering a practical compromise for most laboratories between experimental feasibility and model accuracy.

Together, these findings establish a foundation for quantitative developmental modeling in *D. melanogaster*, providing tools that will facilitate cross-study comparability and improve interpretation of nutritional stress responses. As holidic diets for *D. melanogaster* are not yet fully resolved, the development of a standardized, chemically defined diet would further strengthen the robustness of such models by reducing inter-study variability and enabling more precise nutritional manipulations. This work underscores the value of combining classical model organisms with modern mathematical approaches to advance predictive nutrition and developmental biology.

## Figures and Tables

**Table 1:** Table of ***t_mid_*** parameters extracted from all three primary functions for all sucrose concentrations.

*t_mid_*	0 M	0.15 M	0.3 M	0.45 M	0.6 M	0.75 M	0.9 M	1.05 M	1.2 M	1.35 M	1.5 M
Gompertz	121.7	119.8	124.4	140.2	153.3	159.6	199.8	215.7	260.7	275.0	271.5
Logit	123.5	122.8	130.2	152.8	165.8	172.2	207.7	228.5	266.5	282.1	277.7
Dose–Response	122.2	120.3	125.0	140.9	154.2	160.5	201.1	217.0	262.9	276.7	273.5

**Table 2:** Table of ***s_dvp_*** parameters extracted from all three primary functions for all sucrose concentrations.

*s_dvp_*	0 M	0.15 M	0.3 M	0.45 M	0.6 M	0.75 M	0.9 M	1.05 M	1.2 M	1.35 M	1.5 M
Gompertz	0.043	0.036	0.029	0.023	0.016	0.016	0.015	0.014	0.010	0.014	0.011
Logit	0.106	0.088	0.073	0.055	0.038	0.037	0.052	0.045	0.040	0.056	0.044
Dose–Response	20.98	16.91	13.98	12.27	9.25	10.12	12.07	11.45	9.97	14.94	11.95

**Table 3:** Table for comparison of the bilinear secondary function fit for all three primary functions.

Primary Model	*x_c_*		nRMSE		R^2^	
	*s_dvp_*	*t_mid_*	*s_dvp_*	*t_mid_*	*s_dvp_*	*t_mid_*
Gompertz	0.5810	0.5241	5.92	5.92	0.9864	0.9733
Logit	0.5588	0.5244	8.70	8.70	0.8815	0.9732
Dose–Response	0.5935	0.6224	7.96	7.96	0.9535	0.9753

**Table 4:** Table for AIC values for both linear and bilinear fitting across all primary functions and both parameters.

Model	Gompertz		Dose–Response		Logit	
	*s_dvp_*	*t_mid_*	*s_dvp_*	*t_mid_*	*s_dvp_*	*t_mid_*
Linear	−114.24	63.92	26.23	64.14	−89.23	59.67
Bilinear	−139.68	57.77	10.84	58.05	−110.49	57.06
ΔAIC	25.44	6.15	15.39	6.09	21.26	2.61

**Table 5:** Table for comparison of fit by sampling frequency using the Gompertz primary function and bilinear secondary function.

Sampling Freq./Day	*x_c_*		nRMSE		R^2^	
	*s_dvp_*	*t_mid_*	*s_dvp_*	*t_mid_*	*s_dvp_*	*t_mid_*
Three	0.5810	0.5241	5.92	5.18	0.9864	0.9733
Two	0.5919	0.5185	7.02	5.23	0.9799	0.9726
One	0.3185	0.5239	11.18	5.49	0.9390	0.9699

## Data Availability

Dataset available on request from the authors.

## Supplementary Materials

The following supporting information can be downloaded at https://doi.org/10.5281/zenodo.18705853, Figure S1: Logit transformation for all sucrose concentrations with linear regression.

## Abbreviations

The following abbreviations are used in this manuscript:

BDSC	Bloomington Drosophila Stock Center
KDSC	Kyoto Drosophila Stock Center
EAV	Egg-to-Adult Viability
HSD	High-Sugar Diet
DR	Dose–Response
VBA	Visual Basic for Applications
ANOVA	Analysis of Variance
AIC	Akaike Information Criterion
GRG	Generalized Reduced Gradient
nRMSE	Normalized Residual Mean Standard Error
